# Complex Regional Pain Syndrome. A Comprehensive Review on Neuroplastic Changes Supporting the Use of Non-invasive Neurostimulation in Clinical Settings

**DOI:** 10.3389/fpain.2021.732343

**Published:** 2021-09-21

**Authors:** Andrea Zangrandi, Fannie Allen Demers, Cyril Schneider

**Affiliations:** ^1^Noninvasive Neurostimulation Laboratory (NovaStim), Quebec City, QC, Canada; ^2^Neuroscience Division of Centre de Recherche du CHU of Québec, Université Laval, Quebec City, QC, Canada; ^3^Faculty of Medicine, Université Laval, Quebec City, QC, Canada; ^4^Department Rehabilitation, Université Laval, Quebec City, QC, Canada

**Keywords:** complex regional pain syndrome (CRPS), non-invasive neurostimulation techniques, rTMS, rPMS, tDCS, TENS, maladaptive plasticity, chronic pain

## Abstract

**Background:** Complex regional pain syndrome (CRPS) is a rare debilitating disorder characterized by severe pain affecting one or more limbs. CRPS presents a complex multifactorial physiopathology. The peripheral and sensorimotor abnormalities reflect maladaptive changes of the central nervous system. These changes of volume, connectivity, activation, metabolism, etc., could be the keys to understand chronicization, refractoriness to conventional treatment, and developing more efficient treatments.

**Objective:** This review discusses the use of non-pharmacological, non-invasive neurostimulation techniques in CRPS, with regard to the CRPS physiopathology, brain changes underlying chronicization, conventional approaches to treat CRPS, current evidence, and mechanisms of action of peripheral and brain stimulation.

**Conclusion:** Future work is warranted to foster the evidence of the efficacy of non-invasive neurostimulation in CRPS. It seems that the approach has to be individualized owing to the integrity of the brain and corticospinal function. Non-invasive neurostimulation of the brain or of nerve/muscles/spinal roots, alone or in combination with conventional therapy, represents a fertile ground to develop more efficient approaches for pain management in CRPS.

## Introduction

Complex regional pain syndrome (CRPS) is a rare debilitating disorder characterized by severe and persisting pain affecting one or more limbs. Signs and symptoms are disproportionate owing to the inciting event and include spontaneous and/or movement-induced pain, sensory impairment (allodynia, hyperesthesia), autonomic dysregulation (changes in skin temperature and/or color, abnormal sweating), and motor abnormalities (joint stiffness, tremor, dystonia, and muscle weakness). The inciting event is usually traumatic, such as fracture, surgery outcome, sprain, or contusion, but in ~10% of cases, the precipitating cause remains unknown. CRPS is divided into two main categories based on the absence (type I, 90% of cases) or presence (type II) of nerve lesion at the periphery (1). A third type (“Not Otherwise Specified, or “NOS”) includes patients who do not fulfill the diagnosis criteria, but whose signs and symptoms cannot be better explained by another diagnosis (2). Also, people who were diagnosed only at a later stage when some of the symptoms were resolved can enter the NOS category (although retrospective inspection of medical history shows that they would have fulfilled all criteria for CRPS diagnosis if only they had been assessed at an earlier stage). Due to the variety and complexity of its symptoms and the initial lack of recognition as a disease, CRPS was historically referred to different names [e.g., reflex sympathetic dystrophy, RSD; algodystrophy; causalgia; shoulder-hand syndrome; etc., refer to Merskey (3)]. The 1994 International Association for the Study of Pain (IASP) adopted the appellation of CRPS and affined the diagnosis by establishing specific descriptive criteria. The latter was then improved by the “Budapest Criteria” (refer to Table 1) (2), which are in use even today to diagnose CRPS (4, 5).

**Table 1:** The “Budapest Criteria” for complex regional pain syndrome (CRPS) diagnosis^*^.

1. Continuing pain, which is disproportionate to any inciting event
2. Must report at least one symptom on three of the four following categories (clinical diagnosis) OR in all four (research purpose): Sensory hyperesthesia and/or allodynia Vasomotor: temperature asymmetry and/or skin color changes and/or skin color asymmetry Sudomotor/edema: edema and/or sweating changes and/or sweating asymmetry Motor/trophic: decreased range of motion and/or motor dysfunction (weakness, tremor, dystonia) and/or trophic changes (hair, nail, skin)
3.Must display at least one sign at the time of evaluation in two or more of the following categories (clinical criteria and research purpose): Sensory: evidence of hyperalgesia (to pinpricks) and/or allodynia (to light touch and/or temperature sensation and/or deep somatic pressure and/or joint movement) Vasomotor: evidence of temperature asymmetry (>1^°^C) and/or skin color changes and/or asymmetry Sudomotor/edema: evidence of edema and/or sweating changes and/or sweating asymmetry Motor/trophic: evidence of decreased range of motion and/or motor dysfunction (weakness, tremor, dystonia) and/or trophic changes (hair, nail, skin)
4. There is no other diagnosis that better explains the signs and symptoms

*Taken from Harden et al. (2)*.

* *Diagnosis of CRPS requires to meet all four criteria*.

Complex regional pain syndrome mostly occurs in the age range of 40–70 years (median of 46 years), three to four times more frequently in women (6) and rarely in children (<10% of cases, usually in early adolescence) (7). CRPS worldwide prevalence varies from 5.5 to 26.2 per 100,000 persons per year (8, 9). The upper limb is more often affected (almost 60% of cases) than the lower limb and most cases resolve within the first year (10). But CRPS evolves into a chronic condition in 15–20% of cases, hindering daily life activities and overall quality of life and preventing 31% of these cases to be back to work 2 years after the onset of symptoms (11–15).

To date, the pathophysiology of CRPS remains largely discussed as multifactorial (16). Peripheral sensitization, dysregulation of the autonomic nervous system, and immune dysfunction are known to contribute to the occurrence and development of the syndrome. However, the prevalence and intensity of each mechanism involved can vary between patients and over time, thus laying the stress on the difficulty to treat CRPS and the need for individualization of therapeutic approaches (16, 17). A growing line of research points out that autonomic and sensorimotor disturbances should be viewed as a manifestation of underlying plastic changes that occur in the central nervous system (CNS) (18–20) and which might be also responsible for the evolution of CRPS into a chronic condition.

The present review discusses the use of non-pharmacological non-invasive neurostimulation techniques in CRPS, with regard to CRPS physiopathology, brain changes underlying chronicization, conventional approaches to treat CRPS, current evidence, and mechanisms of action of peripheral and brain stimulation.

## Overview of CRPS Physiopathology

### Peripheral Changes and Central Sensitization

The inciting trauma of CRPS is usually responsible for the inflammation and the immune cascade that trigger the proliferation of connective tissue cells associated with contracture and of keratinocytes that produce inflammatory cytokines; the inflammatory cytokines activate osteoblasts and osteoclasts responsible for the formation and resorption of the bones. This results in less bone density and sensitization of peripheral nociceptors in CRPS, i.e., a lower pain threshold. Precisely, some C-fibers (nociceptive afferents), which usually only transmit nociceptive information from periphery to spinal cord, begin to produce inflammatory neuropeptides (e.g., P-substance); these neuropeptides activate mast cells that release in turn chemical mediators associated with the acute phase symptoms, such as the edema, the skin red coloring and warmth, or hair growth (21, 22). It follows oxidative stress for the patient in the acute phase, as denoted by a higher number of oxygen free and hydroxyl radicals in the saliva and serum (23). At the chronic stage (symptoms present for 6 months and more), pro-inflammatory factors are still present, but it is reported that the inflammatory profile (presence, among others, of interleukins 1 and 6 in the cerebrospinal fluid and interleukins 1, 2, 4, and 7 in blood samples) is different than during the acute phase (symptoms from <6 months; the presence of interleukins 8 and TNFα receptors I and II in the blood) (24). Neurogenic inflammation is also reported in parallel with CNS changes and reciprocal influences are suspected, likely the former influencing the latter in the acute phase and the reverse in the chronic phase (25). For example, at a spinal level, sustained neuropeptide signaling and inflammatory mediators induce persistent central sensitization, which could contribute to the chronicization of pain symptoms (26, 27).

It is noteworthy that cutaneous innervation seems affected even in type-1 CRPS (no nerve lesion) as reflected by lower axonal density (28), lower C-fiber and Aδ-fiber density, and changes in hair follicles and sweat glands innervation (29). In that vein, it was suggested that a minimal distal nerve injury (not detectable) could be the initial trigger for the cascade of events leading to CRPS (16, 28), thus likely explaining why some people do not recall any inciting trauma of their CRPS.

### Dysregulation of the Autonomic Nervous System

CRPS has been considered for a long time as hyperactivity of the autonomic nervous system. This was because of the changes in skin color, temperature, and sweating, and because people were diagnosed with CRPS, only if symptoms were reduced by a stellate ganglion block or by a sympathetic block of the lumbar chain (1, 5, 30). Whether the autonomic nervous system is involved in CRPS pathophysiology is controversial, some authors have reported sympathetic dysfunction in the acute phase and its normalization over 3 months (31, 32), others reported a normal activity or an increase (1, 33–35). This warrants studies on that topic because dysregulation of the autonomic nervous system may at least contribute to state changes (warm vs. cold limb) that cannot be only due to local inflammation (30, 36).

### Immune Dysfunction

In the last decade, research has revealed that antibodies (e.g., of adrenergic and cholinergic receptors) could be present in the serum samples of people with CRPS. This suggests that the immune system could contribute to CRPS chronicization (37–39). Research in this field is booming and the upcoming evidence ought to be considered in future reviews.

## Understanding Brain Changes in CRPS

**Figure 1:**
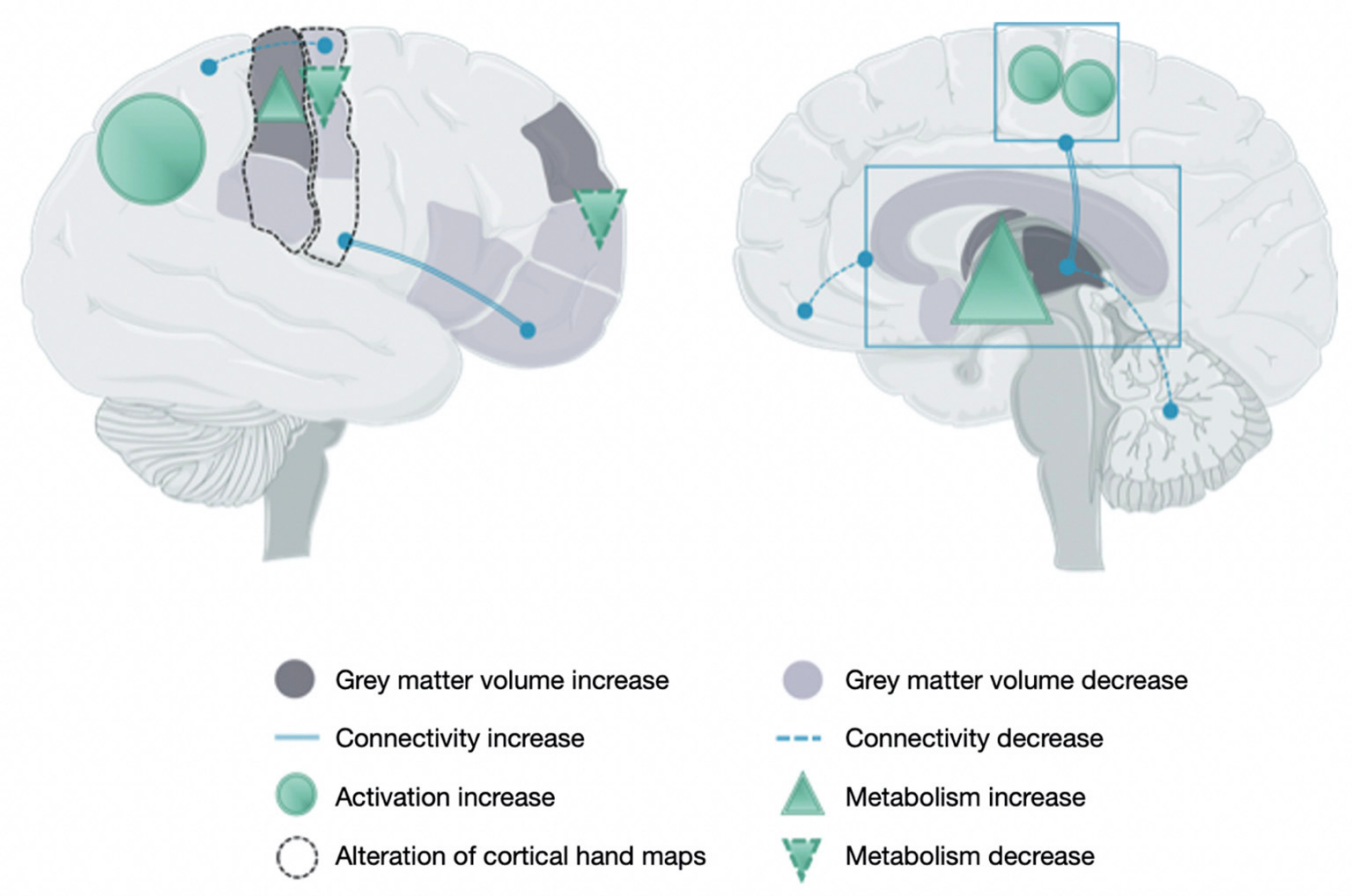
Brain changes in people with a complex regional pain syndrome. This figure illustrates the main data reported in Table 2 owing to the increase or decrease of gray matter volume, connectivity, activity, and metabolism and the alteration of maps related to hand sensorimotor function.

This section deals with the neuronal maladaptive plasticity reported in CRPS and the neuroanatomical and functional changes studied by neuroimaging techniques and transcranial magnetic stimulation (TMS), respectively. Most changes in CRPS are presented in Table 2 and are illustrated in Figure 1.

**Table 2:** Brain changes reported in CRPS.

**Changes of volumes and maps (decrease ↓ or increase ↑)**
↓ Gray matter volume in right anterior insula, OFC, right ventral PFC, CC, inferior PL, SMA, nucleus accumbens, putamen (40–43)
↑ Gray matter volume in the M1 contralateral to CRPS hand, dorso-medial PFC, right hypothalamus, bilateral dorsal putamen, choroid plexus (42, 44)
= or ↓ Extent of CRPS hand maps in the contralateral S1 and in M1 (18, 45–51)
↑ Shifting of CRPS hand map in the contralateral S1 (18)
**Changes of connectivity, activation, metabolism**
**Alteration or decrease (↓) or no change (=)**
↓ Default mode network (52–54)
↓ Connectivity to sensorimotor cortices (41)
↓ Metabolism in the M1 and dorsal PFC (55)
↓ Connectivity between M1 and SPL in the hemisphere contralateral to CRPS side (41)
↓ Connectivity between ventro-medial PFC and basal ganglia (40)
↓ Thalamic perfusion (56, 57)
↓ Connectivity between putamen and cerebellum (43)
↓ Opercular activation during painful stimulation (58)
↓ Pain and sensory threshold via sensitization of *N*-methyl-D-aspartate (glutamate) receptors (59)
**Increase (↑) or no change (=)**
↑ Activation of M1 and SMA during movement (60)
↑ Activation of M1 and SMA at rest (41)
↑ Metabolism bilaterally in S2, mid-anterior and posterior CC, PC, PPC, cerebellum, right posterior insula, and thalamus (55)
= or ↑ Amplitude and frequency of SSEP in the contralateral S1 hand area in response to stimulation on the CRPS side (45, 49)
↓ Suppression of SSEP by paired-evoked paradigm bilaterally (61, 62)
= Peak latency of SSEP (18, 46, 48, 49)
= Peak strength of SSEP (46, 62)
↑ Connectivity between ventro-medial PFC and insula (40)
↑ Connectivity between putamen and pre-post-central gyri (43)
↑ Activation of PPC during painful stimulation (58)
**Changes of Neurophysiological Outcomes (decrease ↓ or no change = or increase ↑)**
↓ Reactivity of M1-related 20-Hz rhythm to tactile stimulation (49, 63)
↓ SICI (23, 50, 64)
↓ LAI (65)
= SAI (65, 66)
= PAS (65)
= Cortical silent period (65, 67)
= RMT (64, 65, 68, 69)
= or ↓ MEP amplitude (64, 65, 67–69)
= or ↑ ICF (64, 65)
↑ I-wave facilitation (23)

*Acronyms of neurophysiological outcomes. SICI, short-interval intracortical inhibition; LAI, SAI, long-/short-afferent inhibition; PAS, paired associative stimulation; RMT, resting motor threshold; ICF, intracortical facilitation; I-wave, indirect wave corresponding to indirect activation of corticospinal cells (at the cell body, not directly at the axons) by transcranial magnetic stimulation*.

*Acronyms for structures. OFC, orbitofrontal cortex; PFC, prefrontal cortex; CC, cingulate cortex; PL, parietal lobule; SMA, supplementary motor area; M1, S1, primary motor and somatosensory cortex, respectively; SPL, superior parietal lobe; S2, secondary somatosensory cortex; PC, parietal cortex; PPC, post-parietal cortex; SSEP, somatosensory-evoked potential*.

### Neuronal Maladaptive Plasticity and Relation to NMDA Receptors

Neuronal plasticity is the capacity of neurons to modulate the efficacy of their synaptic connections with other elements of the CNS (neurons, glial cells). Long-term potentiation (LTP) and long-term depression (LTD) characterize, respectively, the increase and the decrease of synaptic strength. LTP and LTD act via, e.g., isotopic receptors of glutaminergic *N*-methyl-D-aspartate (NMDA), which works as gates for massive inflows of calcium ions in the post-synaptic neuron when previously depolarized (70). The duration of these changes (LTP or LTD) can be influenced by some neuromodulators, such as dopamine, serotonin, acetylcholine, norepinephrine (71–73). That is, the more often synaptic circuits are used, the higher will be the LTP. Thus, the more often pain pathways are activated, the lower will be the threshold to trigger pain messages. In chronic pain, the NMDA receptors lead to the activation of sensory and nociceptive pathways at a lower threshold of peripheral stimuli (a change referred to as “central sensitization”) (74). In CRPS, some studies showed positive results after the administration of an NMDA-antagonist, such as ketamine, either alone (75–77) or in combination with other medications (78). This explains why it was suggested that glutamate NMDA receptors could play a pivotal role in brain maladaptive plasticity in CRPS (59).

### Neuroimaging Studies of Brain Volumes, Activation, and Connectivity

#### Brain Volumes

Changes in CRPS include a decrease in the gray matter volume in the right anterior insula, orbitofrontal cortex (OFC), right ventromedial prefrontal cortex (PFC), cingulate cortex, putamen, sensorimotor cortices, and parietal areas (40–43), and an increase in the gray matter volume in the two dorsal putamen, right hypothalamus, dorsomedial PFC, contralateral primary motor cortex, and choroid plexus (42, 44).

#### Primary Somatosensory Cortex (S1)

Except in one study (45), most magnetoencephalography (MEG), electroencephalography (EEG), or functional MRI (fMRI) data in CRPS confirmed a significant shrinking of S1 hand representation in the hemisphere contralateral to the painful side, as compared to the unaffected hand or pain-free subjects (18, 46–50). One study denoted that the center of gravity of the S1 hand area was shifted to the lip area (18). EEG-recorded amplitudes of somatosensory evoked potentials (SSEPs) following median/ulnar nerve stimulation on the CRPS side showed that the S1 hand area was more responsive to peripheral signal than on the unaffected side or in pain-free people (49), i.e., hyperexcitability but without any change of peak timing (18, 46, 48, 49). Some fMRI studies reported a smaller activation and weaker blood-oxygen level-dependent signal (BOLD) in the CRPS-related S1 area as compared with the other side (46, 79) or to pain-free subjects (80), but one study did not find any between-hemisphere difference (58). Somatosensory excitability was assessed by SSEP using the paired-pulse evoked suppression paradigm. This technique requires the application of two asynchronous stimulations of the median nerve at the level of the wrist, with the expectation that the amplitude of the second SSEP in S1 is significantly smaller than the first. Results showed a marked bilateral reduction of cortical disinhibition in specific tasks, as compared with that of pain-free subjects, thus supporting the dysfunction of somatosensory circuits (61, 62).

#### Motor Areas

The activation of the primary motor cortex (M1) and supplementary motor area (SMA) recorded by fMRI during finger tapping with the CRPS-affected limb was shown to be increased bilaterally but more markedly on the ipsilateral side (60). The technique of arterial spin labeling was used to test the motor-resting neural activity and it was found that blood perfusion in M1 and the SMA was increased in people with chronic CRPS (41). Also, the technique of positron emission tomography with F-fluorodeoxyglucose (FDG-PET) tested that the metabolism of M1 and dorsal PFC contralateral to the CRPS-affected side was decreased as compared to that in pain-free people (55). Two MEG studies investigated the 20 Hz rebound of M1 in response to somatosensory stimulation, which reflects the increase of M1 excitability after a period of suppressed activity due to the somatosensory stimulation. In people with CRPS, 20-Hz oscillations did not adapt properly in response to tactile (49) and noxious (63) stimuli, thus suggesting the alteration of M1 inhibition processes.

#### Non-motor Areas

fMRI recordings during painful stimulation in people with CRPS denoted an increase (either contralateral to the site of stimulation or bilateral) of the responses in the posterior cingulate cortex, in parallel with a decrease of the posterior opercular cortex (58). Several studies found a bilateral increase of the responses in somatosensory cortices, cingulate cortex, parietal cortex, cerebellum, as well as in the right insula and the right thalamus (55). Perfusion was also decreased in the part of the thalamus contralateral to the affected limb (56, 57).

#### Inter-structure Connectivity

Diffusion-tensor imaging (a technique using MRI-recorded direction of water within the myelinated fibers to reconstruct brain tractography) helped detect the increase of connectivity between the ventromedial PFC and insula, and between the putamen and pre-/post-central gyri (43) and the decrease of connectivity between the ventromedial PFC and basal ganglia (40) and between putamen and cerebellum (43). Precisely, the involvement of the basal ganglia in the physiopathology of CRPS was recently hypothesized (81). In support, it was shown that basal ganglia activation to nociceptive stimuli was increased in children (82, 83) and adults with CRPS (58) and that the functional linking between the intraparietal sulcus and caudate nuclei was bilaterally altered in people with CRPS (52). Resting-state fMRI studies also brought about evidence of the alteration of the default mode network in CRPS (52–54).

### TMS Studies of Brain Functional Integrity

Transcranial magnetic stimulation is a reliable tool widely used to study M1 mapping and to characterize markers of M1 and corticospinal excitability in CRPS (84). Some TMS outcomes were reported to be different in CRPS and others unchanged as compared to pain-free people.

#### Cortical Organization

Mapping of M1 representation by single-pulse TMS in people with type-1 CRPS showed that the affected hand had a smaller M1 representation than the unaffected with a center of gravity more variable but not significantly different between sides or compared with that in pain-free people (51).

#### M1 Inhibition

TMS paradigms enable investigation of the different mechanisms of M1 inhibition, and CRPS studies showed that some inhibitory processes could be altered and others not. Paired-pulse TMS of M1 at inter-stimulus intervals below 4 ms showed that the short-interval intracortical motor inhibition (SICI, depending on GABA_A_ receptors activity) (85) was reduced either in both the hemispheres as compared to that in pain-free individuals (64, 68) or only in M1 contralateral to CRSP side (23, 50, 86). Of note, the cortical silent period following a motor-evoked potential (MEP) (superimposed on background isometric contraction) is a different mechanism of M1 inhibition that depends on the GABA_B_-receptors, and which was shown to be comparable between sides in CRPS and to pain-free subjects (65, 67). Also, the long-afferent inhibition (LAI), which investigates sensorimotor integration, i.e., the inhibition of TMS-MEP by sensory afferents volley triggered by electrical stimulation of a peripheral nerve (85), was shown to be reduced in M1 contralateral to the affected hand (65). However, at shorter inter-stimulus intervals aiming at testing the short-afferent inhibition (SAI), the MEP reduction by median nerve stimulation was unchanged as compared to that in pain-free subjects (65, 66). In addition, the paired associative stimulation (PAS), e.g., 180 pairs of nerve electric stimulation and TMS of M1, induced the same sensorimotor plasticity (MEP increase) in CRPS as in pain-free subjects (65). Thus, circuits connecting S1 and M1 seem to work properly in CRPS and may not explain the differences in M1 inhibitory function and plasticity.

#### M1 Facilitation

TMS studies showed controversial findings in CRPS for M1 facilitation. Indeed, paired-pulse TMS of M1 at inter-stimulus intervals over 10 ms showed that the intracortical motor facilitation (ICF, depending on NMDA glutamatergic receptors) could be comparable between sides and to that in pain-free subjects (64) and significantly increased in the hemisphere contralateral to the CRPS side (65).

#### M1 and Corticospinal Excitability

Among other TMS outcomes in CRPS that were unchanged between hemispheres or as compared to pain-free people are the resting motor threshold (RMT) and the amplitude of the MEP tested at 120% RMT (64, 65, 68, 69). RMT is the minimal TMS intensity required to evoke five MEP ≥50 μV out of 10 successive trials in the target muscle at rest and it informs on the basic transsynaptic M1 excitability. The MEP amplitude informs on the corticospinal excitability and it depends on the volume of M1 tissue responding to TMS and on the synchronicity of descending volleys to excite the alpha-motoneurons in the spinal cord. Of note, the same authors showed that MEP amplitudes were either unchanged in CRPS (68) and bilaterally decreased (67) as compared to those in pain-free subjects (refer to Table 2).

### Clinical Significance of Brain Changes

Brain changes in CRPS are extensive and they underlie multiple brain areas, and maladaptive neuronal plasticity has been evidenced as a primary cause of chronicization (17, 87). Pain intensity was reported to be positively correlated with volume changes of the left posterior hippocampus and left amygdala (42) and negatively correlated with volume changes of the bilateral dorsolateral PFC, putamen, and other areas associated with pain processing (41–43). Also, the shrinkage of hand S1 representation increased with the intensity of pain and the presence of hyperalgesia (88) and the motor threshold of the M1 hand area was significantly lower (higher M1 excitability) in the presence of allodynia (68). Most importantly, some abnormal brain changes were found to be reversed, even normalized, concomitantly to the improvement of symptoms and CRPS resolution (17, 88, 89). CRPS chronicization being related to maladaptive brain changes, it is thus legitimate to propose that improvement of the condition in individuals depends on the normalization of these brain changes.

## Are Treatments Adapted to Brain Changes in CRPS?

The previous sections deciphered that neuroanatomical and functional brain modifications in CRPS can be of clinical significance and rely on the somatosensory, motor, and emotional pathways, in one or both hemispheres (refer to Table 2). Data are, however, sometimes controversial, likely due to the fact that some studies tested the acute stage, whereas others the chronic. Also, the clinical significance of brain changes remains unclear, i.e., whether they are specific to CRPS symptoms or shared with other chronic pain conditions. Thus, it is not known whether a conventional treatment that reduces a symptom influences brain maladaptive plasticity, and in other words, whether a treatment actually heals the cause or the consequence. In the latter case, improvement of the condition may only be transient, and the condition will not be resolved due to the persistence of abnormal brain functioning.

The conventional care and follow-up of CRPS are multidisciplinary with pharmacological interventions (often a combination of molecules) (90), local anesthetic sympathetic blockade (91–93), ketamine injections (75, 76, 94, 95), physical or occupational therapy (96, 97), and psychological support (13, 98). However, there is almost no clinical evidence to support these treatments (10, 90, 99), being CRPS often refractory to any intervention. Also, there is no randomized clinical trial published yet to defend the multidisciplinary approach. Overall, CRPS literature remains scarce on a treatment influencing brain plasticity with clinical significance. Conventional treatments that influence sensory integration, such as rehabilitation, may be sometimes of concern because they are based on intensive movement training but people with CRPS can feel pain only by the thoughts of moving the painful part (100, 101), thus creating additional discomfort that could even worsen brain changes. The question is thus whether conventional treatments in CRPS are appropriate and sufficient to normalize brain changes and improve the condition sustainably. For example, S1 and M1 map distortion in CRPS can alter sensorimotor integration as already shown in other pain conditions (e.g., phantom limb pain) (16): people with CRPS take a longer time to recognize the laterality of their affected hand (102) and this leads to a mismatch between sensory information and movement, thus hindering motor control and generating pain. Only an approach nurturing the brain with sensory information from the affected side, to improve sensorimotor control and without creating pain, could contribute to normalize S1 and M1 neuroplasticity and decrease CRPS severity. This has never been addressed by studies using conventional treatment regimens.

The way CRPS is treated should be revisited. Researchers should test the approaches that influence sufficiently the neuroplasticity at the origin of the improvement of the condition. The next section deals with the potential and current evidence of non-invasive neurostimulation to normalize maladaptive brain changes. It is noteworthy, however, that one size does not fit all, i.e., people experience pain differently and respond to treatment differently, thus the same treatment may not be efficient for everyone. It is suggested that individualized protocols of neurostimulation should be developed on the basis of individual brain changes in CRPS.

## Non-Invasive Neurostimulation in CRPS

The use of non-invasive neurostimulation techniques for the management of pain is based on its potential to influence the neuronal plasticity related to the condition (brain changes exposed in the previous sections). These techniques include cortical and peripheral repetitive transcranial magnetic stimulation [rTMS, repetitive peripheral magnetic stimulation (rPMS)], transcranial direct current stimulation (tDCS), and transcutaneous electrical nerve stimulation (TENS). This section presents the rationale for plasticity and the different techniques of non-invasive neurostimulation, the current evidence in the CRPS, and proposes research and clinical prospective.

### Brain Plasticity Influenced by Non-invasive Neurostimulation

Non-invasive neurostimulation can influence cerebral plasticity and reverse maladaptive neural changes either directly by brain stimulation or indirectly via ascending pathways following peripheral stimulation of nerves, muscles, or spinal cord (72, 103–106). These techniques can be potentially implemented into interdisciplinary approaches that are precisely aimed at promoting the central reorganization at the origin of pain reduction. Compared to other invasive treatments, neurostimulation techniques offer multiple advantages. They are painless, particularly with the use of magnetic stimulation (rTMS, rPMS), and do not have side effects (or limited ones such as transient headaches for rTMS and tDCS). Especially, they can be used as add-ons to rehabilitation exercises. Indeed, neurostimulation can normalize the maladaptive brain plasticity responsible for CRPS chronicization, and this influence on plasticity primes and potentiates the effects of the task-oriented rehabilitation, thus making it possible to go beyond the gains already reached and plateaued (107). It is known, furthermore, that patients with CRPS often experience kinesiophobia or fear of movement (101). This said, rPMS of muscles that mimic the contraction/relaxation mechanisms and triggers movements of the CRPS limb with any pain could help reduce kinesiophobia and all the psychological stress surrounding the attempt to move, thus easing at the end the compliance to therapy and its success (106, 107).

### The Different Techniques

#### Cortical and Peripheral Repetitive Magnetic Stimulation (rTMS, rPMS)

Repetitive magnetic stimulation consists of the administration of painless magnetic pulse trains above the brain, e.g., M1 or dorsal PFC (rTMS, T for transcranial), or at the periphery, e.g., over nerve or muscle (rPMS, P for peripheral). The manipulation of the stimulation parameters, such as the frequency (from 0.1 up to 50 Hz), train duration, inter-train interval, coil positioning over a cortical or peripheral target, enables modifying of the actual net after-effects in the neural tissues beneath the coil, i.e., depolarization or hyperpolarization/depression [for details, refer to Pell et al. (73), Beaulieu and Schneider (108)]. In clinical pain studies, rTMS is usually applied over M1 at subthreshold intensity (below the intensity eliciting a muscle response via the corticospinal pathway). The after-effects (LTP-like excitation or LTD-like inhibition) can last from minutes to several hours, depending on the protocol and the task tested and can induce changes of excitability and function in remote areas. Long-lasting rTMS-induced analgesic effects likely rely on LTP/LTD-like mechanisms (refer to the previous section on central sensitization) via an influence on glutamatergic networks (109).

rPMS is commonly applied over a spinal root, nerve, or muscle belly at a suprathreshold intensity to trigger muscle contraction (72). It is hypothesized that it may recruit proprioceptive afferents directly by the depolarization of sensory fibers terminals and indirectly via the induction of repeated contractions and joint movements (108). Also, due to minimal recruitment of nociceptive receptors (the magnetic pulse bypasses skin without resistance), it is painless and the proprioceptive message mediated to the brain is not contaminated by cutaneous information. Thus, rPMS mimics the contraction/relaxation process of one muscle or a group of muscles, and the pure proprioceptive information generated is coherent with the appropriate motor control to influence sensorimotor plasticity at the origin of motor improvement or pain reduction (108). In support, it is shown in motor disorders or in chronic pain that rPMS influences the cortical markers with clinical significance (110).

#### Transcranial Direct Current Stimulation

tDCS is administrated by means of two electrodes (the anode and the cathode) fixed on the scalp. Many studies have shown a greater reduction of pain when the anode is positioned above M1, as compared to S1 or the dorsolateral PFC (111, 112). The cathode is always on the forehead, supraorbital area, contralateral to M1 stimulated. M1 stimulation by tDCS may activate corticospinal and corticothalamic projections which in turn influence the activity of regions of the diencephalon, brain stem, and spinal cord involved in pain modulation mechanisms (113, 114). Specifically, studies show that the effectiveness of tDCS in relieving chronic pain and maintaining effects depends on key stimulation parameters, such as electrode position (anodal M1 montage), stimulation intensity (2 mA), duration (20 min), and the number of weekly sessions (111, 112).

#### Transcutaneous Electrical Nerve Stimulation

TENS can be applied at a high frequency (HF > 50 Hz) with subthreshold intensity (no muscle contraction) or at a low frequency (LF < 10 Hz) with suprathreshold intensity (producing muscle contraction) (115). In humans, both protocols can reduce chronic pain by the generation of somatosensory inputs but their respective mechanisms of action seem different owing to different after-effects due to different frequencies used (116). Also, low-intensity conventional TENS can have maximal analgesic effects homotopically, i.e., on the stimulated side, whereas high-intensity TENS can induce spatially diffused analgesic effects. It has also been shown that only high-intensity TENS produced long-lasting changes in S1 and M1 areas and in their connectivity to vmPFC, which is part of the pain inhibition descending system (116). This activation of the pain inhibition systems promotes the release of endogenous opioids, thus explaining the diffuse analgesic effects (117–119).

### Current Evidence in CRPS

Fourteen studies have been published to date on the use of non-invasive neurostimulation in CRPS, either alone, or combined with other therapies. Table 3 details these rTMS, tDCS, rPMS, and TENS studies.

**Table 3:** Studies with noninvasive neurostimulation in CRPS.

**References**	**Study type**	**Population**	**Intervention**						
			**Stimulation protocol**	**Control**	**Number of sessions**	**Site**	**Parameters**	**Scales and time of testing**	**Outcomes reported**
Bilgili et al. (120)	Double-blinded, placebo-controlled, randomized trial	CRPS type I (*N* = 30) Exp = 15 Sham = 15	TENS + standard physical therapy	Sham stimulation + standard physical therapy	15 Sessions, frequency/week not reported	Active electrode on the dorsal aspect of the forearm, passive electrode on the dorsal aspect of hand	100-Hz TENS (50–100 ms pulse duration) at intensity below the discomfort threshold, 20 min	VAS, LANSS, DN-4, ROM, edema size, functional capacity with hand dynamometer and DHI	Reduction of pain, edema, and fingers ROM
Bodenheim and Bennett (121)	Case report	SA Exp = 1	TENS	na	24 Sessions (3 sessions/week, 8 weeks)	Acupuncture points	20-Hz TENS at intensity adjusted to patient tolerance (100-μs pulse width), 60 min	Clinical evaluation of pain and physical outcomes	Reduction of pain, recovery of ankle ROM, increase of bone stock, and reversal of atrophy
Gaertner et al. (122)	Open-label and non-randomized study	CRPS type I and II (*N* = 21) Exp = 6 Exp = 15	iTBS + rTMS iTBS + rTMS	na na	1 Session 5 Sessions (1 session/day, 5 days)	Contralateral M1 Contralateral M1	iTBS at 70% RMT (5-Hz bursts of 3 pulses at 50 Hz, 2 s ON/8 s OFF, total = 600 pulses) followed immediately by 10-Hz rTMS at 80% RMT (10-s trains, 30-s inter-train interval; total = 2,000 pulses). Total = 2,600 pulses per session	VAS, at baseline, then after the single or the 5 sessions and 2 weeks after	Significant pain reduction after 1 session and 1-week post-treatment; however, no group differences were present
Houde et al. (123)	Case report	CRPS type I Exp = 1	Anodal tDCS Anodal tDCS + TENS	na na	5 Sessions (1 session/day, 5 days) 10 Sessions (1 session/day, 5 days, repeated after 6 months)	tDCS on contralateral M1, TENS over painful area	2-mA tDCS and 3-Hz TENS (400 μs), 25 min	VAS; at baseline, after 15 min of each intervention, after 6 months from tDCS + TENS only	tDCS + TENS slightly reduced pain intensity and unpleasantness
Kesler et al. (124)	Cohort study	RSD (*N* = 10) Exp = 10	TENS + home-based physical therapy	na	Various depending on the patient (4 sessions/day, multiple days)	Over vascular supply of affected extremity	Intensity adjusted to comfort, 60 min. No other information provided	Clinical evaluation of pain and physical outcomes	*N*=7 with complete remission within 2 months
Krause et al. (67)	Cohort study	CRPS type I (*N* = 22) Exp = 12 Control = 10	rPMS	Healthy subjects	1 Session	Over C7/C8	20-Hz rPMS at 120% RMT; 10 trains of 10 s each, inter-train interval not reported; Total = 2,000 pulses over ~10 min	Cortical and spinal MEP, contra-and-ipsilateral cortical silent period; pre-/post-rPMS testing	Less effective input to the motor cortical system
Lagueux et al. (125)	Randomized parallel single blind study	CRPS type I (*N* = 22) Exp = 11 Control = 11	Anodal tDCS + graded motor imagery	Sham stimulation + graded motor imagery	14 Sessions (1 session/day, 5 days/week for 2 weeks, 1 day/week for 4 weeks)	Contralateral M1	2-mA tDCS of 20 min	Pain perception, quality of life, kinesiophobia, pain catastrophizing, anxiety, mood; at baseline, at 6 weeks of treatment and 1 month after the end of treatment	No added value of tDCS combined with GMI for reducing pain
Leo (126)	Case report	RSD Exp = 1	TENS	na	2 Sessions (1 session/day, 22 days apart)	Bilaterally at acupuncture points	4-Hz TENS at intensity below pain threshold, 30 s for each point	Pain and right upper extremity ROM; at baseline and after each session	Reduction of pain and increased ROM at painful, improvements still present at 3 months
Picarelli et al. (127)	Double-blind, placebo-controlled, two-arm, randomized trial	CRPS type I (*N* = 23) Exp = 12 Sham = 11	rTMS + best medical treatment	Sham stimulation	10 Sessions (1 session/day, 5 days/week, 2 weeks)	Contralateral M1	10-Hz rTMS at 100% RMT; 25 trains of 10 s each, 60-s inter-train interval; total = 2,500 pulses over ~29 min	VAS, MPQ, SF-36, HDRS; at baseline, then daily during the 10 sessions and 1 week/3 months after the last session	Reduction of pain and improvement of affective aspects only during the period of stimulation
Pleger et al. (128)	Cohort study	CRPS type I Exp = 10	rTMS	Sham stimulation	1 Session	Contralateral M1	10-Hz rTMS at 110% RMT; 10 trains of 1.2 s each, 10-s inter-train interval; total = 120 pulses over ~2 min	VAS; baseline, 30 s after, then 15/45/90 min after the stimulation	Pain reduction at 30 s with lowest VAS score at 15 min
Richlin et al. (129)	Case report	RSD Exp = 1	TENS	na	30 Sessions (3 sessions/day, 10 days)	Proximal electrode over the right femoral triangle, distal electrode over the dorsum of the right foot	40-Hz TENS at intensity below discomfort threshold, 80-μs pulse width, 30 min	Pain, ROM, thermography, skin temperature; at baseline, 5 days after the beginning of treatment, 2 days later, and 4 weeks from the beginning	Reduction of hyperalgesia, increased ROM, complete pain relief after the treatment
Robaina et al. (130)	Cohort study	RSD Exp = 26	TENS	na	Various depending on the patient (2–5 sessions/day, multiple days)	Painful area or proximal area next to painful area or nerve trunk	80-120-Hz TENS at intensity at parasthesia threshold (50–200 μs pulse width), 30 to 60 min depending on the patient	VAS, MPQ; at baseline and follow-up over 10–36 months	*N*= 20 / 29 with good/excellent pain reduction
Schmid et al. (131)	Case report	CRPS type not specified Exp = 1	Anodal tDCS + sensorimotor hand training	Sham stimulation	1 Session	Contralateral M1	Anodal tDCS for 20 min. No other information provided	Specific sensorimotor hand training, VAS; pre- and post-tDCS testing	Pain reduction and improved performance on ST
Stilz et al. (132)	Case report	RSD Exp = 1	TENS	na	2 Weeks, number of sessions not reported	Proximal electrode over the right femoral triangle, distal electrode over the right foot dorsum	50-Hz TENS at 3.5 mA, no other information provided	Clinical evaluation of pain and physical outcomes	Reduction of pain, hyperesthesia, edema and cyanosis. Pain was still absent after 1 month

*Acronyms of population. RSD, Reflex Sympathetic Dystrophy; SA, Sudeck's Atrophy; CRPS, Complex Regional Pain Syndrome*.

*Acronyms of intervention. TENS, transcutaneous electric current stimulation; tDCS, transcutaneous direct current stimulation; rTMS, repetitive transcranial magnetic stimulation; iTBS, intermittent theta burst stimulation*.

*Acronyms of scales and outcomes. ROM, range of motion; VAS, visual analog scale; MPQ, McGill Pain Questionnaire; SF-36, 36-Item Short Form Survey; HDRS, Hamilton Rating Scale for Depression; ST, sensorimotor hand training; Clinical evaluation: therapist judgment without scales; na, not available*.

#### rTMS

Three studies tested the after-effects of rTMS in people with CRPS: two studies focused on type I (128) and the third on mixed types I and II CRPS (122). All the three administrated rTMS over M1 were contralateral to the CRPS hand. The first two studies used 10 Hz rTMS in a single session with 10 patients or 10 sessions with 12 patients, one time a day for 10 days in a row (46, 128). Precisely, Pleger et al. (128) reported that pain intensity could be reduced after one rTMS session (as measured on the visual analog scale or VAS), as compared to sham stimulation, with the VAS scores being the lowest at 15 min after the end stimulation, but back to baseline at 45 min. One study (127) applied rTMS as an add-on intervention of a standard pharmacological and rehabilitation treatment for 10 consecutive sessions (10 days in a row). Of note, the pharmacological and rehabilitation treatment was first administrated over a month before adding on rTMS. The authors reported a reduction of pain (scores of VAS and McGill Pain Questionnaire or MPQ) and improvements of affective and emotional scores (SF-36 and Hamilton Depression Scale) during the period of rTMS treatment, but the effects had vanished at follow-ups of 1 week and 3 months. The third study (122) was conducted in a mixed cohort (CRPS types I and II). The authors used an open-label and non-randomized design to investigate the after-effects of the priming of 10 Hz rTMS by intermittent theta burst stimulation (iTBS). The protocol of iTBS (5 Hz bursts of three pulses delivered at 50 Hz) was delivered at an intensity of 70% of the RMT and was followed immediately by the 10 Hz rTMS (10 s trains with 30 s inter-train interval) delivered at 80% RMT with the coil guided by real-time neuronavigation. A decrease in the pain, VAS scores were reported immediately after the end of the stimulation and 2 weeks after. Of note, the magnitude of pain reduction was similar between patients having undergone a single session (*n* = 6) and those having been enrolled in five sessions one time a day (*n* = 15).

#### rPMS

One study used rPMS in CRPS (67). Ten series of 10 s of 20 Hz rPMS at 120% of spinal RMT were applied over the cervical nerve roots innervating muscles of the painful area. The authors reported that the amplitudes of MEP to TMS of M1 were smaller on both sides in CRPS than in pain-free participants. The after-effects of rPMS in this study were limited to the lengthening, in pain-free participants only, of the duration of the contralateral and ipsilateral cortical silent periods, which informs on the level of M1 and interhemispheric inhibition, respectively. Unfortunately, this study did not collect any clinical outcomes.

#### tDCS

Three studies investigated the after-effects of anodal tDCS applied over M1 contralateral to the CRPS hand, two case studies (131), and one randomized parallel single-blind study (125). Of note, these studies used tDCS as an add-on of sensorimotor training (131), TENS over the painful area (123), and graded motor imagery (125). Schmid et al. reported that anodal tDCS + sensorimotor training reduced pain intensity and improved the pattern identification during ST, as compared to sham tDCS + sensorimotor training (131). The second study (123) reported that anodal tDCS + TENS one time a day a day for 5 consecutive days slightly reduced pain intensity and unpleasantness, as compared to tDCS alone. Lagueux et al. tested tDCS + graded motor imagery in 11 people with CRPS type I (125). Precisely, the participants underwent 6 weeks of graded motor imagery, and anodal tDCS of M1 was added one time a day for 5 days in a row in the first 2 weeks of graded motor imagery, then, one time a week for the remaining 4 weeks of graded motor imagery. The authors reported that this did not reduce pain more than in the control group of 11 other patients having undergone sham tDCS + graded motor imagery with the same parameters (125). Of note, it has never been reported either that protocols of tDCS alone could improve pain management in CRPS (112).

#### TENS

TENS after-effects in CRPS pain management have been described in numerous interesting case reports and case series since the late 1970s, both in children and adults. However, robust data-evidence-based studies are missing and the efficacy of TENS has not yet been established. Current evidence is limited by the case report designs, the large variety of protocols employed or the missing details in the protocol, the heterogeneous cohorts of patients, and the lack of appropriate control conditions in most cases. However, given the high acceptance and safety of this device, it is almost always worthwhile to consider TENS as part of a multidisciplinary approach (133). For example, three case studies in children aged 10, 6, and 3.5 years old, respectively (126), and one case report in a 43-year-old woman (121) reported that TENS applied over acupuncture points or painful areas, at low or high frequencies and for one or several sessions, decreased pain, hyperesthesia, hyperalgesia, edema, cyanosis if any, and, in parallel, improved the range of motion (ROM) at the painful joint. Two other series of cases used various stimulation protocols between children and with limited details provided in the articles: TENS coupled with home-based physical therapy reduced pain symptoms in 9/10 cases with complete remission within 2 months in 7/10 cases (124) and TENS alone reduced pain in 20/29 cases (130). More recently, a randomized clinical trial tested 100 Hz TENS as an add-on to a standard physical therapy program (contrast bath, whirlpool bath, and physical exercise) in 15 people with type-1 CRPS. The authors showed that 15 sessions of TENS + standard physical therapy program reduced pain scores and edema and increased the second-to-third fingers ROM more than in a group of 15 other patients who underwent sham TENS + standard physical therapy program. It was concluded that the addition of TENS to standard physical therapy programs significantly contributed to clinical recovery in CRPS (120).

### Research and Clinical Prospective

Non-invasive neurostimulation techniques have already been reported to influence neurophysiological markers in various chronic pain conditions, such as fibromyalgia (134, 135), neuropathic pain (136–140), lower back pain (107, 141–143), deafferentation pain (144), or phantom limb pain (145). Evidence is slowly piling up and the work from Moisset et al. (109) in chronic pain reviewed the analgesic effects of rTMS of M1 or of dorsolateral PFC and related this pain reduction to the changes of corticospinal excitability that can last for weeks. In a recent systematic review (146), the use of high-frequency rTMS over M1 was acknowledged level A of evidence in neuropathic pain, level B in CRPS, and the use of high-frequency rTMS over the left dorsolateral PFC was acknowledged level B of evidence in the control of pain. It is thus surprising that, despite promising data in other chronic pain conditions, only a few randomized clinical studies tested these techniques in CRPS (112). A possible explanation is that CRPS is a rare syndrome characterized by a large variabilty of clinical profiles, thus making it difficult to run larger studies with randomized placebo-controlled designs. In fact, two systematic reviews rated with very low quality of evidence on the therapeutic effects of non-invasive neurostimulation in CRPS on pain intensity, but this may be due to small sample size and short follow-up (99, 147).

Future research is warranted to better determine whether non-invasive neurostimulation, alone or combined with other treatments, is efficient to reduce CRPS severity. To this end, studies ought to clearly identify if there are responders and non-responders to one or another technique if some data or variables collected at baseline can be predictors of responsiveness, and if all brain changes detected are specific to CRPS, or commonly related to chronic pain, or, for example, a consequence of the limb non-use, i.e., not directly related to pain. Due to the high variability of CRPS profiles, it is expected that not all people will respond to non-invasive neurostimulation to the same extent. It will thus be crucial to customize an individual approach owing to brain changes and the integrity of the corticospinal system.

## Conclusion

Knowledge of CRPS neurophysiopathology has evolved rapidly in the last decades, with more evidence of neural changes involvement in the chronicization, the symptoms and the resistance to conventional treatment. However, literature on non-invasive neurostimulation trials to influence these neural changes remains scarce. This is despite evidence from other pain conditions of sustained pain decrease and function improvement in parallel with a resolution of maladaptive neural plasticity. The current review lays the stress on the fact that non-invasive neurostimulation of the brain or of nerve/muscles/spinal roots, alone or in combination with other treatments, represents a fertile ground for further investigations on more efficient interventions in CRPS management.

## Author Contributions

FAD, AZ, and CS wrote the manuscript. AZ and CS edited the manuscript. All authors listed have made a substantial, direct and intellectual contribution to the work, and approved it for publication.

## Funding

The work was supported by the Chronic Pain Network funded by the strategy for patient-oriented research (SPOR) of the Canadian Institutes of Health Research (CS's lab). AZ was supported by a doctorate studentship from the Center Thématique de Recherche en Neurosciences (CTRN-Université Laval, Québec). FAD was supported by a master's studentship for health professionals from the Fonds de Recherche du Québec-Santé (FRQS).

## Conflict of Interest

The authors declare that the research was conducted in the absence of any commercial or financial relationships that could be construed as a potential conflict of interest.

## Publisher's Note

All claims expressed in this article are solely those of the authors and do not necessarily represent those of their affiliated organizations, or those of the publisher, the editors and the reviewers. Any product that may be evaluated in this article, or claim that may be made by its manufacturer, is not guaranteed or endorsed by the publisher.
